# The plasmonic BTO-on-SiN platform – beyond 200 GBd modulation for optical communications

**DOI:** 10.1038/s41377-025-02116-1

**Published:** 2025-12-16

**Authors:** Manuel Kohli, Daniel Chelladurai, Laurenz Kulmer, Tobias Blatter, Yannik Horst, Killian Keller, Michael Doderer, Joel Winiger, David Moor, Andreas Messner, Tatiana Buriakova, Clarissa Convertino, Felix Eltes, Yuriy Fedoryshyn, Ueli Koch, Juerg Leuthold

**Affiliations:** 1 ETH Zurich, Institute of Electromagnetic Fields, Zurich, Switzerland; 2 Ligentec SA, Ecublens, Switzerland; 3 Lumiphase AG, Stäfa, Switzerland

**Keywords:** Optoelectronic devices and components, Photonic devices

## Abstract

An integrated photonics platform that offers high-speed modulators in addition to low-loss and versatile passive components is highly sought after for different applications ranging from AI to next-generation Tbit/s links in optical fiber communication. For this purpose, we introduce the plasmonic BTO-on-SiN platform for high-speed electro-optic modulators. This platform combines the advantages provided by low-loss silicon nitride (SiN) photonics with the highly nonlinear barium titanate (BTO) as the active material. Nanoscale plasmonics enables high-speed modulators operating at electro-optical bandwidths up to 110 GHz with active lengths as short as 5 µm. Here, we demonstrate three different modulators: a 256 GBd C-band Mach-Zehnder (MZ) modulator, a 224 GBd C-band IQ modulator – being both the first BTO IQ and the first IQ modulator on SiN for data communication – and finally, a 200 GBd O-band racetrack (RT) modulator. With this approach we show record data rates of 448 Gbit/s with the IQ modulator and 340 Gbit/s with the MZ modulator. Furthermore, we demonstrate the first plasmonic RT modulator with BTO and how it is ideally suited for low complexity communication in the O-band with low device loss of 2 dB. This work leverages the SiN platform and shows the potential of this technology to serve as a solution to combat the ever-increasing demand for fast modulators.

## Abstract (web summary)

This plasmonic BTO-on-SiN platform enables 256 GBd high-speed data transmission. It can further operate beyond 200 GBd for IQ and O-band racetrack modulators within highly compact device footprints.

## Graphical abstract

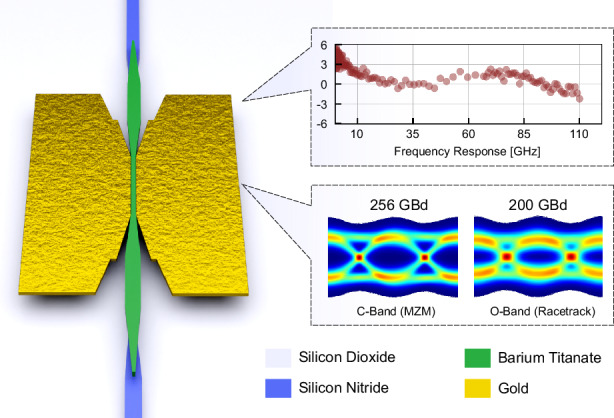

## Introduction

A high-speed photonic platform that offers a combination of electro-optic modulators with low-loss passive components is essential in many fields where light must be manipulated with electrical signals. For instance, such a combination can be crucial to further advance Tbit/s optical communication links^1^, photonic quantum computing^2^, input/output interfaces to cryogenic environments^3–6^, disaggregated AI systems^7^, microwave photonics^8,9^, and optical computing^10,11^. To meet the increased total traffic demands and complexity in systems, the ideal integrated optical platform should offer high speed operation, a compact footprint, enable operation across the largest possible spectral range, and be able to handle high input powers.

There exists a variety of different platforms and approaches for integrated high-speed modulators. An important metric to demonstrate the potential of a technology is the maximum achievable signal bandwidth. Demonstrations over 200 GBd in the C-band include BTO plasmonics with symbol rates up to 216 GBd^12^, POH up to 256 GBd^13^, and TFLN up to 260 GBd^14^. Most of these demonstrations were realized by intensity modulation and direct detection (IM/DD) schemes. Coherent transmission, on the other hand, is ideal for high data rates in long-haul communications, where complex modulation formats with IQ modulators allow the encoding of information in both phase and amplitude of the light. Examples of IQ modulators operating beyond 100 GBd include plasmonic-organic hybrid (POH) experiments showing single-polarization net-data rates of 790 Gbit/s at 160 GBd^15^, silicon photonics with polarization multiplexed (PMUX) net-data rates of 1 Tbit/s at 105 GBd^16^, thin-film lithium niobate (TFLN) with PMUX net-rates of 1.96 Tbit/s at 130 GBd^17^, and InP with PMUX net-rates of 2.03 Tbit/s at 192 GBd^18^. To achieve such high numbers, most demonstrations utilize probabilistic shaping of high-order modulation formats (up to 100QAM and more) in addition to PMUX, where data is encoded on carriers with orthogonal polarization. In contrast, the O-band is ideally suited for low-complexity, and therefore low-cost applications with IM/DD, due to low fiber dispersion^19^. Recently, directly modulated lasers have been demonstrated to achieve 256 GBd in the O-band^20^, which is an attractive option for intra-data center communications. Integrated solutions with high symbol rates include TFLN^21,22^ modulators operating at 210 GBd and InP absorption modulators at 256 GBd^23^.

Although impressive demonstrations, high-speed modulators could generally benefit from the advanced passive performance, scalability, ultra-low loss^24,25^, transparency across a large spectral range^26,27^, and the ability to handle high input power due to negligible two-photon absorption and relatively low stimulated Brillouin scattering^28^ offered by SiN photonics. Its advantages have brought forward impressive demonstrations such as frequency comb generation^29,30^, on-chip amplifiers^31^, quantum sources^32^, and lasers^33,34^. Yet, SiN does not offer an electro-optic effect to modulate the light at high frequencies. Among all nonlinear effects, the Pockels effects is particularly interesting as it offers a pure phase modulation^35^. Within interferometric configurations, one can also implement amplitude or intensity modulation. With the demonstration of ultra-low losses in TFLN^36^, it has been developed into a highly versatile platform with a Pockels effect for many different applications^37,38^. In contrast to TFLN, however, SiN is CMOS compatible and already available on 300 mm wafers as it can be grown directly on silicon photonics. Therefore, significant effort has been dedicated to integrating Pockels-effect modulators onto the SiN platform. Examples include PZT-based modulators reaching 40 GBd^39^, SiN-loaded TFLN reaching 80 GBd^40,41^, and heterogeneously integrated TFLN reaching 80 GBd^42–45^. SiN loaded BTO has been leveraged to demonstrate ultra-low-power tuning^46^. By utilizing a plasmonic slot waveguide, it is possible to achieve modulation in a most compact footprint and very high speeds. More recently, we introduced plasmonic BTO-on-SiN and demonstrated 216 GBd in the C-band^12^. BTO has emerged as a viable candidate for integrated electro-optic modulators^47–50^. It offers one of the largest Pockels coefficients among known materials^51^, it is suited for cryogenic environments^3^, and allows wafer-scale integration with advanced platforms^2,52^. Furthermore, BTO with the combination of plasmonics offers exceptionally good thermal stability up to 250 ° C in addition to longtime stable operation at 90 ° C^53^. It is therefore conceivable that the combination of SiN with BTO gives access to a scalable integrated optical platform that offers highest speed on a compact footprint, with an ability to handle high power across a wide spectrum.

In this work, we show the potential of the BTO-on-SiN integrated optical platform that allows for the implementation of a wide variety of active components targeted for different applications, more specifically short-haul and long-haul communication. For instance, we demonstrate fully integrated Mach-Zehnder (MZ), IQ, and racetrack (RT) modulators. Operation with this BTO-on-SiN platform is shown up to highest speed of 256 GBd and line rates of up to 448 Gbit/s. Such high speed has been enabled by implementing plasmonic metal-insulator-metal active sections that operate up to 110 GHz. The favorable frequency response enables operation with as little as 1.13 V_pp_ at 200 GBd – which by any standard makes it an attractive solution for driverless operation. The plasmonic approach further allows integration of the active components on a most compact footprint, which may be as small as 5, 15 and 17.5 μm for the RT, the MZ and the IQ modulators, respectively. This work demonstrates the first BTO-based IQ modulator and the first IQ modulator on SiN. The IQ modulator is the ideal configuration for long-haul communication. The combination of the low loss SiN passive technology with the resonant BTO-plasmonic RT modulators in O-band, ideally suited for low-cost intensity-modulation/direct-detection scheme, yields devices with low 2 dB on-chip losses. Lastly, we show operation across a large spectral window by demonstrating devices for the 1300 and 1550 nm window. All devices were fabricated on the same chip with the same method, and we thus elevate the plasmonic BTO approach to a modulator platform that can be tailored to the specific application. To tackle the energy efficiency required for new optical engines, we demonstrate that high-speed performance of these modulators is achieved even with low-complexity linear offline digital signal processing (DSP), which could enable energy-efficient real-time processing. The plasmonic BTO-on-SiN platform can reach up to 196 GBd with linear equalization only.

The paper is based in part on work that was initially presented at OFC’24 and CLEO’24 conferences^54,55^.

## Results

### BTO-on-SiN platform

**Fig. 1:**
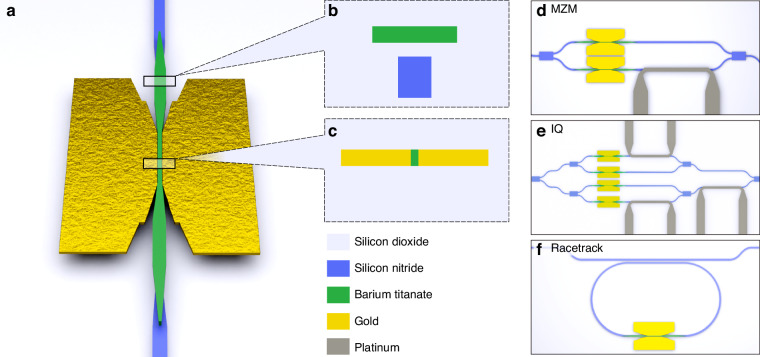
Schematic of the BTO-on-SiN platform. **a** Overview of the high-speed BTO-on-SiN modulator featuring the combined advantages of low-loss SiN, highly nonlinear BTO, and nanoscale plasmonics. The high-speed device consists of a directional coupler from SiN to BTO, a short BTO waveguide (<200 µm in length), a photonic-to-plasmonic converter and a plasmonic waveguide section. **b** Cross-section of the SiN-to-BTO directional coupler with below 0.2 dB/transition loss. **c** Cross-section of the plasmonic BTO waveguide featuring two gold metal plates with BTO in between. Schematics of the different measured modulators with **d** C-band MZ modulator, **e** C-band IQ modulator, and **f** O-Band RT modulator

The BTO-on-SiN platform aims to combine the advantages provided by the low-loss SiN, the nonlinear BTO with one of the highest Pockels effect among known materials, and the nanoscale plasmonics offering highest speed in most compact configurations. A basic building block of a Pockels modulator is the phase shifter. More complex configurations of modulators, such as the MZ and IQ, can be composed of multiple phase shifters. A schematic of the high-speed plasmonic BTO-on-SiN phase shifter, capable of 110 GHz operation, is shown in Fig. 1a. The modulator can be divided into two main parts, the SiN passives used to route the light on the chip, and the phase shifters on the top layers composed of BTO and gold.

The silicon nitride waveguide is fully embedded in SiO_2_ and features a cross-section of 800$\times$800 nm^2^ in the C-band and 600$\times$800 nm^2^ in the O-band. The fiber-to-chip coupling is solved by employing amorphous silicon overlay grating couplers in a simple scheme. By adding a metallic mirror to the same structure, the coupling efficiency is improved from −2.2 dB to −1.4 dB in experiment and from −1.1 dB to −0.44 dB in simulation^56^. The O-band gratings are designed to be dual-polarization with a high efficiency of −2.5 dB in simulation and −3 dB in experiment for coupling both TE and TM polarizations with the same grating^56^. A heater based on a 100-nm-thick and 2-µm-wide platinum strip can be used to control the phase relation in interferometric configurations such as MZ and IQ modulator. This metal is located ~1 µm above the SiN waveguide separated by SiO_2_ cladding. For future improvements in terms of footprint and energy efficiency, the phase tuners could be optimized by increasing the thermal isolation^57^, or replaced for example with solutions based on Pockels effect or on liquid crystals^46,58–60^.

The phase shifters are composed of the following parts: a vertical directional coupler (VDC) to couple the light to the BTO, a photonic-to-plasmonic converter (PPC) to focus the light into the active section, and the plasmonic waveguide. The active phase shifters are composed of a metal-insulator-metal waveguide with gold and BTO to form the plasmonic slot waveguide. To couple light from the SiN into the phase shifters, there are two stages. First, the signal is coupled to a BTO waveguide with a VDC from the SiN waveguide layer, see Fig. 1b. The length of the VDC in the C-band modulators, i.e., the MZ and the IQ modulator, is 80 µm in length. For the O-band RT modulator, it is shortened to 40 µm to achieve a shorter round-trip path for the light in the RT section. In Fig. 1b, the cross-section of the directional coupler is shown. The 800-nm-thick SiN waveguide is tapered from a width of 800 nm to 200 nm, whereas the ~200-nm-thick BTO waveguide located roughly 100 nm above the SiN, is tapered from a width of 150 nm to the single-mode waveguide of 800 nm in the C-band and 600 nm in the O-band. Cutback measurements in the C-band indicate that the propagation loss in the BTO is as low as 4.5 dB/cm and therefore has negligible losses for the short propagation distances of below 200 µm. The transition loss of the VDC is 0.14 dB/transition determined from cutback measurements. In the PPC, light is focused into the plasmonic slot by tapering the BTO waveguide and bringing the metal closer until it touches the BTO to form the plasmonic slot, see Fig. 1c. The working principle of this plasmonic converter is described in our previous work and 3D finite-element (FEM) simulations indicate losses below 1 dB^12^. The measured losses are discussed in the sections below. With the routing done in SiN, this phase shifter can be placed on top of more complicated structures to form different types of modulators. In the following, we discuss the experimental results of three modulator types: the C-band MZ, the C-band IQ, and the O-band resonant RT modulators.

### C-Band MZ modulator

**Fig. 2:**
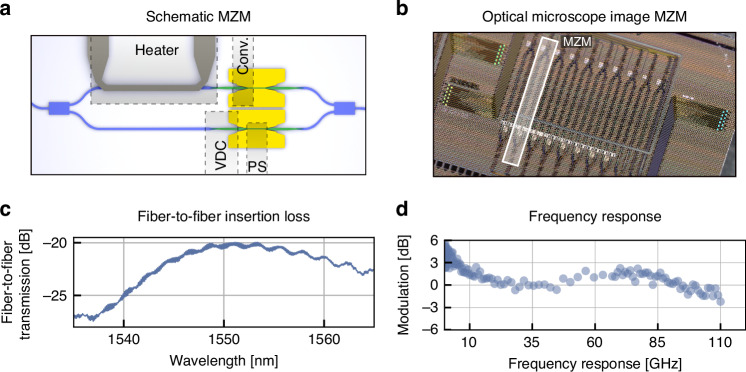
Characterization of C-band MZ modulator. **a** Schematic of the MZ modulator with phase shifters in each arm and a platinum heater to set the operating point of the modulator. **b** Optical microscope image of fabricated MZ modulators. The highlighted area shows a single device. **c** Fiber-to-fiber insertion losses. **d** The initial drop below 10 GHz is inherent to the frequency response of the BTO Pockels effect as seen in plasmonic devices. Yet, the modulator’s frequency response relevant for the high-speed data experiment drops only 3 dB in the range between 10 GHz and 110 GHz

A schematic overview of the C-Band MZ modulator is shown in Fig. 2a. Light is first split into two arms with multi-mode interferometers in SiN. It is then coupled to the plasmonic phase-shifter sections and mapped back to waveguides where they are recombined in a multi-mode interferometer coupler (MMI). Figure 2b shows an optical microscope image of the fabricated device. The plasmonic phase-shifter section is 150 nm wide and 15 µm long. The photonic-to-plasmonic converter is 15 µm long. The fiber-to-fiber insertion losses are shown in Fig. 2c. In the current fabrication run, we found insertion losses (ILs) of −20.3 dB at 1550 nm. Through cutback measurements, we found grating coupler losses of 2.8 dB per coupler, plasmonic propagation losses of 0.5 dB/μm in the 150-nm-wide slot, and 3.5 dB loss per photonic-to-plasmonic coupler. However, simulations and reference measurements indicate that the fiber-to-fiber losses can be ideally as low as 8.1 dB. These lower losses can be split onto 5 dB of losses in the plasmonic section (0.33 dB/µm as derived from measured material properties), 1 dB per PPC (3D FEM simulations, see ref. ^12^), 0.1 dB per VDC transition for a total of 0.2 dB, and 0.44 dB per grating coupler with metallic mirror, see ref. ^56^. There is thus room for fabrication improvement. Particularly, the PPC show much higher losses than anticipated due to fabrication issues.

We measure a ${{\rm{V}}}_{\pi }$ of 3.6 V in the phase shifter or 1.8 V in the Mach-Zehnder configuration in push-pull configuration at DC. The frequency response of the phase shifters in the MZ modulator from 10 MHz to 110 GHz is shown in Fig. 2d. We find a drop-off between the MHz and the lower GHz ranges, leading to a ${V}_{{\rm{\pi }}}$ of 6.4 V at 40 GHz. We extract an effective Pockels effect of ~180 pm/V at 40 GHz by comparing measured and simulated ${{\rm{V}}}_{{\rm{\pi }}}$. The drop is due to the frequency dependence of the Pockels effect in BTO and can be directly observed in a plasmonic configuration^12,48^. The response flattens after the initial drop and is followed by a small resonance around 75 GHz. We attribute this resonance to an LC peaking due to parasitic inductances of the prober set-up and device. The frequency response drops off around 110 GHz. The drop-off can be explained by an RC limit. This occurs due to the capacitance of the modulator and the 50 Ohm source. We expect improvements in the ${V}_{\pi }$ and bandwidth with further optimizations of the device cross section and fabrication. Nevertheless, the BTO plasmonic MZ modulator reaches 110 GHz with a 3-dB drop-off between 10 GHz and 110 GHz, which suffices for highest symbol rates of 256 GBd. Therefore, this modulator is more than capable of being employed in the next generations of Tbit/s links.

### C-Band IQ modulator

**Fig. 3:**
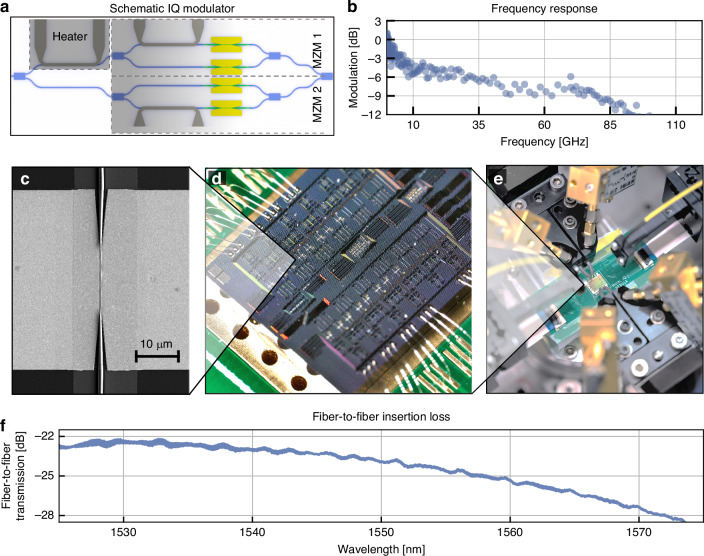
Characterization of C-band IQ modulator. **a** Schematic of the IQ modulator consisting of two MZ modulators. **b** Frequency response of the IQ modulator featuring a cutoff at around 80 GHz. We find a 3-dB drop between 10 and 70 GHz. **c** SEM image of a phase shifter in the IQ modulator. **d** Optical microscope image of the IQ modulators wire bonded to a PCB. **e** Measurement setup for the IQ modulator. The PIC was diced and wire-bonded onto a PCB to contact all DC connections of the IQ modulator. **f** Fiber-to-fiber transmission of the IQ modulator

The schematic of the IQ modulator can be seen in Fig. 3a. It is composed of two parallel MZ modulators constituting the in-phase and quadrature phase modulators. A third platinum heater is added to set the phase difference between the in-phase and quadrature signals. The footprint of a single IQ modulator is 0.75$\times$2.15 mm^2^, dominated by the footprint of the three heaters ($\sim$400$\times$150 µm^2^). The plasmonic sections in this IQ modulator are 17.5 µm in length and 100 nm wide. Figure 3d shows an optical microscope image of the chip containing the IQ modulators. The diced PIC was placed on a PCB. Wire bonds connect the PIC to the PCB to contact the IQ modulators with the DC current sources for the heaters.

The fiber-to-fiber insertion loss of the IQ modulator is shown in Fig. 3f. The modulator features a total insertion loss of 22.5 dB at 1530 nm and 23.9 dB at 1550 nm. Through cutback measurements, we attribute ~2.75 dB to the grating couplers, 12.25 dB to plasmonic losses in the active section (~0.7 dB/µm) in the 100 nm wide plasmonic section and ~ 3dB per photonic-to-plasmonic coupler. Simulations indicate that the losses can be reduced to ~12 dB fiber-to-fiber IL in this configuration. This includes higher losses due to the narrower slot and longer length in comparison to the MZ modulator.

The electro-optic response was measured by applying a sinusoidal signal to the phase shifters in the IQ modulator. Figure 3f shows the electro-optic modulation as a function of frequency. We measure the characteristic drop between MHz and GHz, similarly to the MZ modulator. However, in the IQ modulator, the higher capacitance, due to a longer modulator and a smaller plasmonic gap, limits the bandwidth. The frequency response starts to drop at ~70 GHz. We measure a ${{\rm{V}}}_{\pi }$ of 4 V in the phase-shifter or 2 V in the Mach-Zehnder configuration at DC voltages. The increased ${V}_{\pi }$ in comparison to the MZ modulator could potentially arise from surface effects or dead layers in the BTO due to etching^53^, since the longer length and smaller width of the modulator should result in a lower ${V}_{\pi }$. Additionally, the bandwidth limitations further suggest that wider slots are preferable.

### O-Band RT modulator

**Fig. 4:**
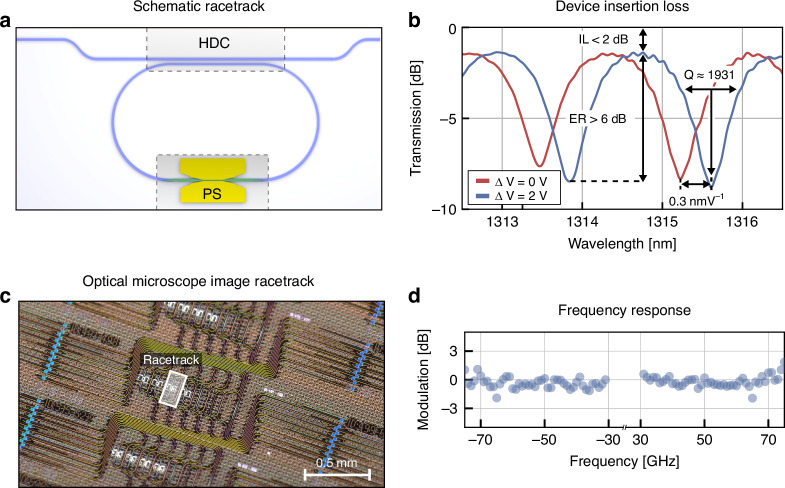
Characterization of O-band RT modulator. **a** Schematic of the O-band RT modulator with a 5 µm long plasmonic phase shifter. **b** On-chip transmission as a function of wavelength and bias voltage. The modulator features a quality factor Q of 1931 and less than 2 dB on-chip loss. **c** Optical microscope image of the fabricated RT. **d** Normalized modulation response in the upper and lower sidebands of the optical carrier in the on-state as a function of frequency

The O-band RT modulator consists of a silicon nitride bus waveguide with a horizontal directional coupler (HDC) to a RT containing a plasmonic phase shifter, see Fig. 4a. Light is coupled from the bus waveguide into the RT. There is constructive or destructive interference between the light in the bus waveguide and the light from the RT. This is dependent on the phase difference accumulated through one round trip. Thus, there are transmission maxima and minima depending on the wavelength. With a phase shifter, the optical path length can be modulated within the RT, changing the spectral position of the minima, respective maxima. Figure 4b depicts the normalized on-chip transmission as a function of wavelength for two different voltages. The red and blue curves have different voltages applied with a $\Delta$V = 2 V. We find fiber-to-fiber transmission loss of −9.4 dB in the center of the O-band at 1310–1315 nm. Measurements of reference waveguides show on-chip device losses of less than 2 dB. The quality factor of the RT modulator is measured to be Q = 1931 with an extinction ratio of more than 6 dB. The free spectral range is measured to be 1.79 nm. By applying a DC voltage to the phase shifter after fully poling the BTO, we measure a tuning efficiency of 0.3 nm/V. This corresponds to a ${V}_{\pi }$ of 3 V at DC. The frequency response of the modulator in the through-state is shown in Fig. 4d. Typically, resonant based modulators suffer from temperature sensitivity. Plasmonic racetrack modulators, however, have shown an improved temperature sensitivity over silicon microring modulators^61^. Furthermore, the combination of plasmonics with BTO has shown stable modulation up to 110 °C in a MZ configuration^53^. A similar temperature sensitivity is expected with this approach due to the small quality factor and small dimensions of the RT modulator.

## Discussion

### Performance in data experiments

The high-speed performance of the modulator is tested with data experiments in the C- and in the O-band for long- and short-haul applications.

**Fig. 5:**
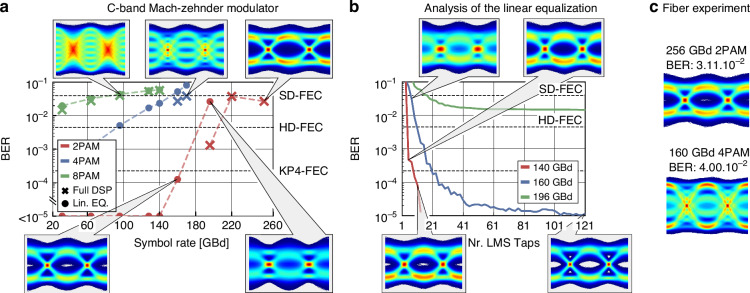
Data experiment of MZ modulator **a** Data experiment results of the MZ modulator. It reaches 256 GBd using 2PAM, 170 GBd using 4PAM, and 96 GBd using 8PAM, while staying below the SD-FEC limit with 20% overhead. With simplified DSP consisting of linear equalization only, we reach 196 GBd below the SD-FEC and 160 GBd below the KP4-FEC limit. **b** BER as a function of the taps in the LMS filter. We compare 140 GBd with the 160 and 196 GBd. Only a small number of taps are required for the 140 GBd signal. Still, a minimum of 3 is necessary to equalize. For the 160 GBd 21 taps allow transmission below the KP4-FEC limit. **c** Eye diagrams of the 400 m fiber transmission using full DSP. In 2PAM, 256 GBd can be transmitted with a BER of 3.11$\cdot$10^−2^. In the 4PAM transmission experiment, the 400 m fiber showed a larger penalty in comparison to back-to-back measurements. 160 GBd was successfully transmitted with a BER of 4.00$\cdot$10^−2^

The C-band MZ modulator operates at symbol rates up to 256 GBd. The measurement setup and DSP chain to achieve this high performance are described in the methods section. The bit-error ratio (BER) as a function of the transmitted symbol rate is shown in Fig. 5a. We reach a maximum symbol rate of 256 GBd using 2PAM with a BER of 2.67$\cdot$10^−2^ with the full DSP consisting of linear and nonlinear equalization. This BER is below the soft-decision forward error correction (SD-FEC) limit with 20% overhead of 4.0$\cdot$10^−2^, see ref. ^62^. For symbol rates up to 196 GBd, the BER is below the hard-decision FEC (HD-FEC) limit of 3.8$\cdot$10^−3^, see ref. ^63^. The results of higher order modulation formats are shown in Fig. 5a in purple and light blue. We achieve a symbol rate of 170 GBd using 4PAM for a BER of 3.75⋅10^−2^.

This corresponds to a maximum line rate with the MZ modulator of 340 Gbit/s. In the case of the 8PAM transmission experiment, 96 GBd is transmitted with a BER of 3.98 × 10^−2^.

Furthermore, the modulator is tested in a fiber-transmission experiment with 400 m fiber length. The eye diagrams of these experiments are shown in Fig. 5c. There is only a small degradation when compared against the back-to-back eye diagrams. In detail, using 2PAM, 256 GBd was transmitted with a BER of 3.10$\cdot$10^−2^, while we transmitted 4PAM 160 GBd with a BER of 4.00$\cdot$10^−2^. We estimate the capacitance of the modulator to be ~30 fF from the frequency response, which leads to an energy consumption of ~10 fJ/bit in the MZ modulator.

The experiments are typically conducted with a full DSP consisting of linear and nonlinear equalization. However, certain applications require simpler complexity, e.g. number of multiplications in the data analysis. This is especially relevant for low-cost and energy-efficient IM/DD links. We thus further analyze the modulators by employing only linear equalization in the DSP chain, specifically with a timing recovery and a feed-forward equalization (FFE) with 21 taps in the IM/DD links. We demonstrate that the BTO-on-SiN modulators can offer up to 196 GBd even in this simpler setting, see Fig. 5a. For symbol rates up to 160 GBd, the BER is below the KP4-FEC limit. Only for higher symbol rates, nonlinear equalization is necessary to compensate for non-idealities in the electrical path. To analyze FFE’s performance with the MZ modulator, the BER of different 2PAM signals is plotted as a function of the number of taps in the FFE in Fig. 5b. With an FFE of less than 10 taps, the MZ modulator can transmit data below the KP4-FEC limit for symbol rates up to 140 GBd. With the 160 GBd signal, the KP4-FEC limit is achieved with only 21 taps.

**Fig. 6:**
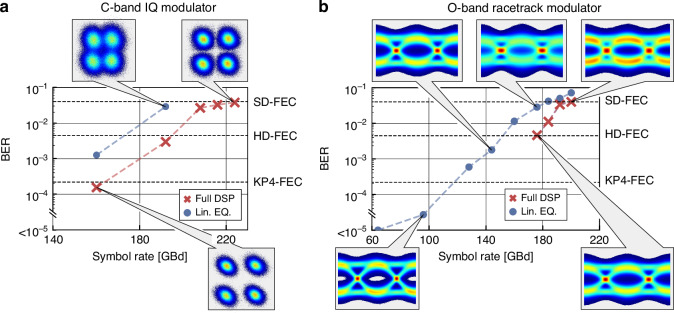
Data experiment of IQ and RT modulator. **a** Data experiment results of the IQ modulator reaching 224 GBd below the SD-FEC, 192 GBd below the HD-FEC and 160 GBd below the KP4-FEC limit using 4QAM. With simplified DSP, data rates up to 192 GBd can be transmitted below the SD-FEC limit. **b** Data experiment of the O-band RT modulator. The crossed values represent the full DSP, whereas the dots depict the simplified DSP. The RT reaches 200 GBd below the SD-FEC with full DSP and 176 GBd with the linear equalization only

With the more sophisticated C-band IQ modulator we show 224 GBd with a BER of 3.79$\cdot$10^−2^ transmission with a coherent setup, see Methods. Figure 6a shows the data experiment and the received constellation diagrams of the C-band IQ modulator. The transmitted 224 GBd in 4QAM with a total line rate of 448 Gbit/s below the SD-FEC limit marks the highest data rate achieved both with BTO as active material on any substrate and with any nonlinear materials on silicon nitride. Similarly to the MZ modulator, simplified DSP with linear equalization only can be employed for symbol rates up to 192 GBd staying below the SD-FEC limit and up to 160 GBd below the HD-FEC limit. Full DSP can be used up to 160 GBd to remain below the KP4-FEC limit.

The resonant RT modulator was operated with 200 GBd in the O-band with a low 2 dB device insertion loss. The results of the data transmission experiment are shown in Fig. 6b. The modulator reaches 200 GBd using 2PAM with a BER of 3.10$\cdot$10^−2^. For symbol rates below 180 GBd, the transmitted signal has a BER below the HD-FEC limit. The same simplified DSP as for the MZ modulator can be employed up to 176 GBd. For rates up to 140 GBd, the BER is below the HD-FEC limit. Lower data rates in comparison to the C-band MZ modulator can be explained by three main reasons. First, the amplifier employed in the O-band has a higher noise figure. Second, the photodetector is optimized for the C-band and has a lower responsivity in the O-band than those at 1550 nm. Thirdly, the optical bandwidth of the resonant modulator is lower than that of the MZ modulator due to the resonant effect. However, this could be solved by optimizing the design. For instance, resonant plasmonic devices with an organic electro-optic material operating in the C-band have already shown operation at 220 GBd employing RT modulators with bandwidths in excess of 100 GHz and presumably 200 GHz^61^. Similarly high numbers may be anticipated with BTO plasmonics by optimizing the plasmonic length, the coupling into the RT, and the total length of the cavity.

We demonstrate a high-speed BTO-on-SiN platform offering beyond 200 GBd data transmission using IM/DD in the C- and O-band and coherent communications in the C-band. All devices were fabricated on the same chip. The potential of this technology is demonstrated in multiple modulators. For instance, the C-band Mach-Zehnder modulator reaches 256 GBd in 2PAM and 170 GBd in 4PAM for a line rate of 340 Gbit/s. This modulator features a ${V}_{\pi }$ = 1.8 V at DC. The high-speed performance allows the employment of simplified DSP consisting only of timing recovery and linear equalization with 21 taps, while reaching 196 GBd. This allows a reduction of DSP complexity and therefore a reduction of energy consumption. We further demonstrate the first BTO-based IQ modulator and the first high-speed IQ modulator on the SiN platform. This modulator reaches 224 GBd 4QAM for a total of 448 Gbit/s line rate. Finally, the high-speed BTO plasmonic phase shifter can be employed in an RT modulator. We demonstrate 200 GBd with an O-band BTO RT modulator featuring a 5 µm long phase shifter and an on-chip loss of less than 2 dB. The same simplified DSP can be employed up to 176 GBd. We show a tuning efficiency of 0.3 nm/V with a quality factor of 1931. These demonstrations show that the BTO-on-SiN platform offers a solution for high-speed electro-optic modulators in short- and long-haul communications.

## Materials and methods

### Fabrication

The modulators in this work were all fabricated on the same chip. The SiN waveguides were produced in a photonic foundry on 8-inch wafers. An oxide cladding was used to cover the waveguides prior to being prepared for the wafer-scaled integration of the BTO active material. The modulators themselves were fabricated on the same die in a back-end-of-the-line (BEOL), chip-scale process on top of the BTO-on-SiN. The BTO was patterned using electron beam lithography. Afterwards, the material was etched to form the directional couplers and the BTO waveguides. The metallization to form the plasmonic slot was conducted with electron beam evaporation and patterns were defined with polymethylmethacrylate (PMMA). Amorphous silicon was deposited using plasma-enhanced chemical vapor deposition (PECVD). The gratings were etched locally using inductively coupled plasma reactive ion etching (ICP-RIE) and the silicon was removed selectively to the materials underneath without damaging the surface. In the next step, the chip was coated with SiO_2_ deposited using PECVD as a cladding. For the heater structures, further metallization steps needed to be added with crossings between metal layers in addition to SiN and BTO waveguides. Approximately 1 µm distance between the individual layers was used to lower loss and parasitic coupling. Connections between different metal layers in addition to openings for contacting were made by photolithography. These structures were etched using ICP-RIE. Finally, the chip was diced into different parts. The chip with the IQ modulators was bonded onto a PCB with a conductive glue and finally wire bonds were made to connect the PCB to the IQ modulators with the DC control voltages.

### Characterization

The cutback measurements were conducted using a tunable laser source in the C-band, respective O-band. An optical power meter was used to track the fiber insertion losses. Cutback measurements allowed an approximation of the losses in the individual components. The DC V_π_ was measured by applying a voltage directly from a small signal source to the modulator and tracking the optical output. Prior to sweeping the voltage, the BTO was fully poled. The high-speed electro-optic bandwidth measurements were conducted by applying a sinusoidal signal to the modulator. This signal was combined using a high-speed bias-tee. Two different methods to generate the signal were used. For measurements up to 70 GHz, an RF source was directly used. The modulation sidebands were tracked with an optical spectrum analyzer. For the measurements from 70–110 GHz, an RF mixer was used to increase the frequency of the RF source. An overlap at 70 GHz allowed to match the signal of the generated signals. For calibration of the setup, the losses in the electrical path were measured by connecting it to an electrical spectrum analyzer. The losses of the probes were taken from the data sheet provided by the manufacturer.

### Data transmission experiment

**Fig. 7:**
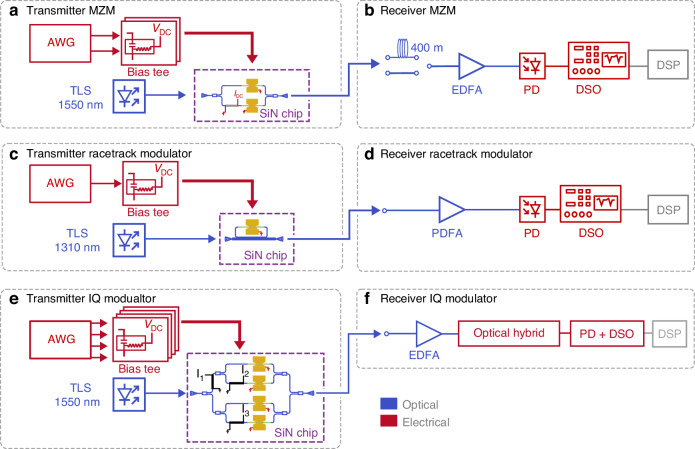
Measurement setup for data experiments. **a**, **b** IM/DD measurement setup for the C-band MZ modulator transmission experiment. **c**, **d** IM/DD measurement setup for the O-band RT modulator. **e**, **f** Coherent measurement setup for the C-band data transmission experiment of the IQ modulator

The measurement setups of the data transmission experiments are shown in Fig. 7. In (a, b), the setup for the MZ modulator measurement is shown, in (c, d) for the RT modulator, and in (e, f) for the IQ modulator.

The MZ modulator was measured in an IM/DD setup. On the transmitter side, see Fig. 7a, a tunable laser in the C-band is coupled to the chip with ~20.3 dBm input power to generate an optical carrier at $\lambda$ = 1550 nm. A periodically repeated signal is generated with an arbitrary waveform generator (AWG). These square-root-raised cosine shaped bit sequences were generated for symbol rates up to 256 GBd. The signal is combined with a DC bias using a high-frequency bias-tee. The biasing is used to pole the domains of the BTO and achieve best modulation efficiency. This voltage value was adapted to a value between 2 V and 3 V for all measurements in this work. On the receiver side, see Fig. 7b, the signal was amplified using an erbium-doped fiber amplifier (EDFA). We recorded the signal with a photodetector and a real-time digital sampling oscilloscope (DSO). We conducted two experiments with the MZ; one is optical back-to-back, and in the other the modulated signal is sent through a 400 m long fiber before the EDFA.

The measurement setup for the IQ modulator, see Fig. 7e, f is optimized for coherent communication. The MZ modulators were operated in push-pull. The carrier was set to $\lambda$ = 1550 nm with ~21 dBm input power to the chip. On the receiver side, an optical hybrid consisting of two photodetectors with a local oscillator was employed after being amplified with an EDFA. The signal was then recorded with a DSO.

The measurement setup for the RT modulator, see Fig. 7c, is similar to the C-band MZ modulator. The laser is exchanged with a tunable laser in the O-band. The carrier was set to $\lambda$ = 1315.7 nm with an input power (in the fiber before the device) of ~13 dBm. The EDFA was exchanged with a praseodymium-doped fiber amplifier (PDFA). A 70 GHz C-band photodetector was used, for which we expect a reduced responsivity in the O-band.

Two different types of DSP were employed for all measurements. The first type is linear equalization only. In the case of the MZ and the RT modulators, this simplified DSP consists of a timing recovery^64^ and a feed forward equalizer (FFE) filter with 21 taps only. For the IQ modulator, an additional carrier recovery was conducted prior to the FFE. The full DSP was adapted for each modulation format and modulator individually. In the case of the MZ modulator, the full DSP consisted of a timing recovery with a T/2-spaced FFE similar to the simplified DSP. This FFE featured 151 taps. The nonlinear equalization was based on a 7-symbol pattern mapping (MAP). Finally, a second T-spaced FFE with 251 taps was applied. In the case of the 8PAM modulation format, the MAP was reduced to 5-symbols and the second FFE was increased to 1001 taps. In the case of the 4PAM, a third-order Volterra was added prior to the 7-symbol pattern mapping. Interestingly, full DSP for the 4PAM signal did not perform much better than the simplified DSP. By increasing the T-spaced FFE to 151 taps, 160 GBd in 4PAM was successfully transmitted below the SD-FEC limit. The full DSP of the RT modulator was identical to the DSP of the 2PAM signal with the MZ modulator. In the case of the IQ modulator, however, the DSP consisted of a carrier recovery, timing recovery, and a FFE with 251 taps. The nonlinear equalization consisted of a 7-symbol pattern mapping with a second FFE of 251 taps. For all modulators, an approximate driving voltage of 1.13 V_pp_ was applied. This value was measured at 200 GBd with electrical back-to-back measurements. Losses of the electrical path were subtracted with values from the datasheets of the individual components at 50 GHz to include the approximate average losses as seen by the electrical signal.

## Supplementary information

Supplementary Information for The Plasmonic BTO-on-SiN Platform – Beyond 200 GBd Modulation for Optical Communications

## Data Availability

The data that support the findings of this study are available from the corresponding authors on reasonable request.
