# Supplementary material for: The plasmonic BTO-on-SiN platform – beyond 200 GBd modulation for optical communications
Source: Light Sci Appl. 2025 Dec 16;14:399. doi: 10.1038/s41377-025-02116-1 (PMC12705825; doi:10.1038/s41377-025-02116-1)
Supplement: Supplementary file 1 — Supplementary Information for The Plasmonic BTO-on-SiN Platform – Beyond 200 GBd Modulation for Optical Communications [file 41377_2025_2116_MOESM1_ESM.docx]

# Supplementary Information for

# The Plasmonic BTO-on-SiN Platform – Beyond 200 GBd Modulation for Optical Communications

Manuel Kohli^1^, Daniel Chelladurai^1^, Laurenz Kulmer^1^, Tobias Blatter^1^, Yannik Horst^1^, Killian Keller^1^, Michael Doderer^1^, Joel Winiger^1^, David Moor^1^, Andreas Messner^1^, Tatiana Buriakova^2^, Clarissa Convertino^3^, Felix Eltes^3^, Yuriy Fedoryshyn^1^, Ueli Koch^1^,
and Juerg Leuthold^1^

^1^ETH Zurich, Institute of Electromagnetic Fields, 8092 Zurich, Switzerland
^2^Ligentec SA, 1024 Ecublens, Switzerland
^3^Lumiphase AG, Laubisrütistrasse 44, 8712 Stäfa, Switzerland

## Comparison of Modulator Technologies

The following table compares the different technologies to our demonstration in key metrics. The focus is put on Pockels-based modulators in addition to Silicon Photonics. The table shows that our approach is among the best in all figure-of-merits apart from loss. Our MZM has an on-chip loss of 14.7 dB (20.3 dB fiber-to-fiber with 2.8 dB per grating coupler). But even these losses are not yet fundamental. Of the 14.7 dB loss, a total of 7 dB is the photonic-to-plasmonic converter, which can be improved with better fabrication. From simulation, the fundamental plasmonic loss is 0.33 dB$\cdot$µm^-1^ $\times$ 15 µm $=$ 4.95 dB. In comparison to TFLN, the plasmonic BTO-on-SiN has three orders of magnitude smaller active area (phase shifter length). Even compared to travelling wave (TW) BTO or PLZT devices, it is two orders of magnitude smaller. In contrast to the plasmonic-organic approach, the active material is solid state and thus reliability concerns at higher temperatures are reduced. Plasmonic BTO modulators have found to be temperature stable for up to 120 degrees in operation and resilient to exposure to up to 250 degrees when put into an oven, see ^1^. For CPO applications, the most critical features are high-bandwidth, small footprint and operation at high temperature. It can be seen, that our Plasmonic BTO modulator - while not being the best in any categories – combines some of the best properties in almost any category.

| Technology | Bandwidth | Footprint* | VpiL | Symbol Rate | Operation at high T | On-Chip Loss | Type of active Material | On-Chip Optical Power | E/bit |
| --- | --- | --- | --- | --- | --- | --- | --- | --- | --- |
| TW-Silicon^2^ | 67 GHz | >6 mm | 30 Vmm | 120 GBd | n/a | 8.5 dB | Solid-state | ~19.5 dBm |  |
| TW-TFLN^3^ | >67 GHz | 4x8 mm^2^ (folded) | 22.5 Vmm | 90 GBd | n/a | n/a | Solid-state | n/a | 0.48 fJ/bit |
| TW-TFLN^4^ | 110 GHz | 23.5 mm | 23.5 Vmm | 130 GBd | n/a | 3 dB | Solid-state | n/a | 1.04 fJ/bit |
| TW-TFLN^5^ | 110 GHz | 23 mm | 23 Vmm | 260 GBd | n/a | 3 dB | Soild-state | n/a | n/a |
| TW-BTO^6,7^ | ~ 30 GHz (40 GHz 6-dB) | 0.4x1.6 mm^2^ | 4.8 Vmm | 128 GBd | n/a | 2 dB | Solid-state | 10 dBm | 9 fJ$\cdot$bit^-1^ |
| TW-PLZT^8^ | 70 GHz | 0.4x2.5 mm^2^ | 5.8-7.0 Vmm | 178 GBd | Yes | 1.5 dB | Soild-state | n/a | n/a |
| Plasmonic-Organic^9^ | >70 GHz | 0.001x0.01 mm^2^ | 0.06 Vmm | 72 GBd | n/a | 7.55 dB | Organics | 8.9 dBm | 25 fJ$\cdot$bit^-1^ |
| Plasmonic-Organic^10^ | >100 GHz | 0.01 mm^2^ | n/a | 256 GBd | n/a | 12 dB | Organics | 3.1 dBm | n/a |
| Plasmonic-Organic^11^ | 997 GHz | 0.01 mm | 0.25 Vmm | - | n//a | 5.6 dB | Organics | -8.9 dBm | n/a |
| Plasmonic-Organic^12^ | n/a | 0.003x0.025 mm^2^ | 0.13 Vmm | 100 GBd | 75° C | 11.2-14.5 dB | Organics | n/a | <2 fJ$\cdot$bit^-1^ |
| Plasmonic BTO | 110 GHz | 0.015  mm | 0.054 Vmm | 256 GBd | Yes** | 14.7 dB | Solid-state | 17.5 dBm*** | 10 fJ$\cdot$bit^-1^ |

* Active area of phaseshifter

**Demonstrated in ^1^
*** 5 dB fundamental plasmonic losses in this configuration

Resonant modulators are a very interesting solution in short-reach communication because of small footprint and small size. Inspired by the Reviewers request of a table, we compiled a second table comparing the performance metrics of resonant modulators. Racetrack modulators typically reduce the losses in comparison to Mach-Zehnder, especially for plasmonic modulators. Our demonstration offers high bandwidth with highest tuning efficiency in comparison to other approaches. Here, tuning efficiency is the shift of the resonance dip with applied voltage.

| Technology | Bandwidth | Q Factor | Tuning Efficiency | FSR | Symbol Rate | On-State Loss | Type of active Material |
| --- | --- | --- | --- | --- | --- | --- | --- |
| SiPh MRM ^13^ | 49 GHz | 3700 | ~16.3 pm$\cdot$V^-1^ * | 5.7 nm | 100 GBd | 0.9 dB | Solid-state |
| SiPh MRM^14^ | 54 GHz | 5200 | n/a | n/a | 130 GBd | n/a | Solid-state |
| SiPh MRM^15^ | >60 GHz** | 4500 | 26.4 pm$\cdot$V^-1^ | n/a | 120 GBd | n/a | Soild-state |
| TFLN^16^ | 30 GHz | 8000 | 7 pm$\cdot$V^-1^ | n/a | 40 GBd | 1.5 dB | Solid-state |
| PZT^17^ | 33 GHz | 2230 | 13.4 pm$\cdot$V^-1^ | 1.7 nm | 40 GBd | n/a | Solid-state |
| PZT^18^ | 24.9 GHz | 8000 | 35.8 pm$\cdot$V^-1^ | 3 nm | 56 GBd | n/a | Solid-state |
| Plasmonic-Organic^19^ | 176 GHz | 700 | 178 pm$\cdot$V^-1^ | 7 nm | 220 GBd | 1.7 dB | Organics |
| Plasmonic-Organic^19,20^ | >100 GHz | n/a | n/a | 4.73 nm | 256 GBd | 1.2 dB | Organics |
| Plasmonic BTO | 70 GHz | 1931 | 300 pm$\cdot$V^-1^ | 1.79 nm | 200 GBd | 2 dB | Solid-state |

## References

1. Messner, A. *et al.* Plasmonic Ferroelectric Modulators. *J. Light. Technol.* **37**, 281–290 (2019).

2. Mohammadi, A., Zheng, Z., Zhang, X., Rusch, L. A. & Shi, W. Segmented Silicon Modulator With a Bandwidth Beyond 67 GHz for High-Speed Signaling. *J. Light. Technol.* **41**, 5059–5066 (2023).

3. Xu, M. *et al.* Attojoule/bit folded thin film lithium niobate coherent modulators using air-bridge structures. *APL Photonics* **8**, 066104 (2023).

4. Xu, M. *et al.* Dual-polarization thin-film lithium niobate in-phase quadrature modulators for terabit-per-second transmission. *Optica* **9**, 61–62 (2022).

5. Mardoyan, H. *et al.* First 260-GBd single-carrier coherent transmission over 100 km distance based on novel arbitrary waveform generator and thin-film lithium niobate I/Q modulator. in *ECOC 2022* Th3C.2 (Optica Publishing Group, 2022).

6. Li, W. *et al.* Thin-Film BTO-Based MZMs for Next-Generation IMDD Transceivers Beyond 200 Gbps/λ. *J. Light. Technol.* **42**, 1143–1150 (2024).

7. Eltes, F. *et al.* Thin-film BTO-based modulators enabling 200 Gb/s data rates with sub 1 Vpp drive signal. in *Optical Fiber Communication Conference (OFC) 2023 (2023), paper Th4A.2* Th4A.2 (Optica Publishing Group, 2023). doi:10.1364/OFC.2023.Th4A.2.

8. Mao, J. *et al.* Ultra-fast perovskite electro-optic modulator and multi-band transmission up to 300 Gbit s−1. *Commun. Mater.* **5**, 114 (2024).

9. Haffner, C. *et al.* All-plasmonic Mach–Zehnder modulator enabling optical high-speed communication at the microscale. *Nat. Photonics* **9**, 525–528 (2015).

10. Kulmer, L. *et al.* Single Carrier net 400 Gbit/s IM/DD over 400 m Fiber enabled by Plasmonic Mach-Zehnder Modulator. *Opt. Fiber Commun. Conf.* (2024).

11. Horst, Y. *et al.* Ultra-wideband MHz to THz plasmonic EO modulator. *Optica* **12**, 325–328 (2025).

12. Heni, W. *et al.* Plasmonic IQ modulators with attojoule per bit electrical energy consumption. *Nat. Commun.* **10**, 1694 (2019).

13. Yuan, Y. *et al.* A 5 × 200 Gbps microring modulator silicon chip empowered by two-segment Z-shape junctions. *Nat. Commun.* **15**, 918 (2024).

14. Chan, D. W. U., Wu, X., Lu, C., Lau, A. P. T. & Tsang, H. K. Efficient 330-Gb/s PAM-8 modulation using silicon microring modulators. *Opt. Lett.* **48**, 1036–1039 (2023).

15. Zhang, Y. *et al.* 240  Gb/s optical transmission based on an ultrafast silicon microring modulator. *Photonics Res.* **10**, 1127–1133 (2022).

16. Wang, C., Zhang, M., Stern, B., Lipson, M. & Lončar, M. Nanophotonic lithium niobate electro-optic modulators. *Opt. Express* **26**, 1547–1555 (2018).

17. Alexander, K. *et al.* Nanophotonic Pockels modulators on a silicon nitride platform. *Nat. Commun.* **9**, 3444 (2018).

18. Liu, G. *et al.* Highly efficient lead zirconate titanate ring modulator. *APL Photonics* **9**, 066111 (2024).

19. Eppenberger, M. *et al.* Resonant plasmonic micro-racetrack modulators with high bandwidth and high temperature tolerance. *Nat. Photonics* **17**, 360–367 (2023).

20. Blatter, T. *et al.* Plasmonic Ring Resonator Modulator Demonstrating IM/DD > 400G per lane. in *European Conference on Integrated Optics* (2024).
